# MicroRNAome profiling in benign and malignant neurofibromatosis type 1-associated nerve sheath tumors: evidences of PTEN pathway alterations in early NF1 tumorigenesis

**DOI:** 10.1186/1471-2164-14-473

**Published:** 2013-07-13

**Authors:** Julien Masliah-Planchon, Eric Pasmant, Armelle Luscan, Ingrid Laurendeau, Nicolas Ortonne, Mikael Hivelin, Jennifer Varin, Laurence Valeyrie-Allanore, Valérie Dumaine, Laurent Lantieri, Karen Leroy, Béatrice Parfait, Pierre Wolkenstein, Michel Vidaud, Dominique Vidaud, Ivan Bièche

**Affiliations:** 1 UMR745 INSERM, Université Paris Descartes, Sorbonne Paris Cité, Faculté des Sciences Pharmaceutiques et Biologiques, 4 avenue de l’Observatoire, 75006 Paris, France; 2 Service de Biochimie et de Génétique Moléculaire, Hôpital Cochin, AP-HP, Paris, France; 3 Département de Pathologie, AP-HP and Université Paris Est Créteil (UPEC), Hôpital Henri-Mondor, Créteil, France; 4 Department of Plastic and Reconstructive Surgery, AP-HP and Université Paris Est Créteil (UPEC), Hôpital Henri-Mondor, Créteil, France; 5 Department of Plastic and Reconstructive Surgery, Hôpital Européen Georges Pompidou, AP-HP, PRES Sorbonne Paris Cité, Université Paris Descartes, Paris, France; 6 Département of Dermatologie, Hôpital Henri-Mondor, AP-HP and EA 4393 LIC, Université Paris Est Créteil (UPEC), Créteil, France; 7 Department of Orthopedic Surgery, Cochin Hospital (AP-HP), Paris, France; 8 Platform of Biological Ressources, AP-HP and Université Paris Est Créteil (UPEC), Hôpital Henri-Mondor, Créteil, France; 9 Laboratoire d’Oncogénétique, Institut Curie, Hôpital René Huguenin, FNCLCC, 92211 Saint-Cloud, France

**Keywords:** NF1, Neurofibromatosis type 1, MicroRNAs, MPNST, Neurofibroma, PTEN

## Abstract

**Background:**

Neurofibromatosis type 1 (NF1) is a common dominant tumor predisposition syndrome affecting 1 in 3,500 individuals. The hallmarks of NF1 are the development of peripheral nerve sheath tumors either benign (dermal and plexiform neurofibromas) or malignant (MPNSTs).

**Results:**

To comprehensively characterize the role of microRNAs in NF1 tumorigenesis, we analyzed 377 miRNAs expression in a large panel of dermal and plexiform neurofibromas, and MPNSTs. The most significantly upregulated miRNA in plexiform neurofibromas was miR-486-3p that targets the major tumor suppressor gene, *PTEN*. We confirmed *PTEN* downregulation at mRNA level. In plexiform neurofibromas, we also report aberrant expression of four miRNAs involved in the RAS-MAPK pathway (miR-370, miR-143, miR-181a, and miR-145). In MPNSTs, significant deregulated miRNAs were involved in *PTEN* repression (miR-301a, miR-19a, and miR-106b), RAS-MAPK pathway regulation (Let-7b, miR-195, and miR-10b), mesenchymal transition (miR-200c, let-7b, miR-135a, miR-135b, and miR-9), *HOX* genes expression (miR-210, miR-196b, miR-10a, miR-10b, and miR-9), and cell cycle progression (miR-195, let-7b, miR-20a, miR-210, miR-129-3p, miR-449a, and miR-106b).

**Conclusion:**

We confirmed the implication of *PTEN* in genesis of plexiform neurofibromas and MPNSTs in NF1. Markedly deregulated miRNAs might have potential diagnostic or prognostic value and could represent novel strategies for effective pharmacological therapies of NF1 tumors.

## Background

Neurofibromatosis type 1 (NF1) is a common autosomal dominant disorder affecting 1 in 3,500 individuals worldwide. Typical clinical features of NF1 include multiple café-au-lait spots, axillary freckling, iris Lisch nodules, and neurofibromas. Neurofibromas are benign peripheral nerve sheath tumors designated as either dermal neurofibromas (DNFs) or plexiform neurofibromas (PNFs). PNFs are regarded as congenital benign tumors that often develop in association with major nerve tracts. About 30 to 50% of NF1 patients develop clinically visible PNFs [1]. Although PNFs are considered as benign tumors, they can be life threatening when they develop deeply and compress internal organs. PNFs are complex tumors, heterogeneous at the cellular level, mainly composed of Schwann cells, which are the likely pathogenic cell type in neurofibromas, together with fibroblasts, mast cells, neurons, vascular elements, and perineurial cells. In contrast to DNFs, PNFs can transform in malignant peripheral nerve sheath tumors (MPNST) in about 10% of the cases. MPNSTs are highly invasive sarcomas that metastasize widely and display a poor prognosis.

NF1 is caused by germline heterozygous loss-of-function mutations in the tumor suppressor gene *NF1* located at 17q11.2. *NF1* encodes neurofibromin, a RAS-GTPase-activating protein (RAS-GAP). Neurofibromin negatively regulates the RAS signalling pathways (i.e. MAPK and PI3K/AKT/mTOR) involved in proliferation, survival, and differentiation. According to the Knudson’s two-hit model, NF1 tumorigenesis results from a somatic mutation disrupting the second functional copy of the *NF1* gene. This complete inactivation of *NF1* induces RAS signaling pathway activation and seems required but not sufficient for tumorigenesis promotion. Rare additional molecular alterations have been described in PNFs, including *CDKN2A/B* locus deletions [2]. In contrast, MPNSTs are characterized by complexe genomic changes including inactivation of *TP53* and *RB1* and amplification of *EGFR*, *HGF*, and *MET*[3]. Expression studies have reported differential expression profile in MPNSTs of genes involved in cell proliferation, apoptosis, invasion, extracellular remodeling and Schwann cell differentiation such as *TP53*, *RB1*, *CDKN2A*, *TWIST1*, *BIRC5*, *TOP2A*, and *SOX9*[4-6]. Finally, transgenic mouse models have provided evidences for *TP53* and *PTEN* implication in PNFs formation and Schwann cells malignant transformation [7,8].

Since their initial discovery in *Caenorhabditis elegans* in 1993 [9], the highly conserved small non-coding RNAs called microRNAs (miRNAs) have been extensively implicated in human physiology and pathology. In the last few years, miRNAs have revealed major roles in regulating critical biological processes such as development, proliferation, differentiation, and apoptosis. MiRNAs aberrant expression has also been characterized in many human cancer types. However, the involvement of miRNAs deregulation in the formation of benign neurofibromas and malignant progression from PNFs to MPNSTs remains largely unknown. Here, we used real-time quantitative reverse transcription-PCR (RT-PCR) assays to quantify the expression of a panel of 377 well-validated miRNAs in a large series of NF1-associated tumors (including nine DNFs, 41 PNFs, and 15 MPNSTs), two normal peripheral nerve samples, and two MPNST cell lines.

## Results

We quantified the expression of 377 miRNAs in nine DNFs, 41 PNFs, and 15 MPNSTs. We also analyzed miRNAs expression in two adult peripheral nerves as a non-tumorigenic control tissue and in two MPNST cell lines (ST88.14 and 90–8) as malignant controls. A significant number (122/377; 32.4%) of miRNAs were below the detection level of the assay (median Ct ≥ 40) in MPNSTs, PNFs, and DNFs and were consequently regarded as “not expressed”. Eighty-four (84/377; 22.3%) miRNAs were considered as detectable but not reliably quantifiable (32 < median Ct < 40) in the three groups of tumors. Thereby, more than half of miRNAs (206/377; 54.6%) were not further analyzed in our study. In each sample, the negative control assay unrelated to mammalian species, ath-miR159a, was not expressed (Ct ≥ 40).

### Unsupervised hierarchical clustering

Unsupervised hierarchical clustering of the 65 NF1-associated tumors, two adult peripheral non-tumorigenic control nerves, and the two NF1-associated MPNST cell lines identified six main subgroups, based on the expression of the 171 miRNAs considered as expressed and reliably quantifiable. One of the subgroup contained 12 of the 15 MPNST samples together with both MPNSTs cell lines (88–14 and 90.8) and no other tumor types. The three remaining MPNST samples (MPNST2, MPNST7, and MPNST9) clustered in a small subgroup of six tumors. Our unsupervised hierarchical clustering discriminated MPNSTs from benign neurofibromas but failed to distinguish between both types of neurofibromas (*i.e.* between DNFs and PNFs). This result mainly reflects that miRNAs expression profile is more deregulated in MPNSTs than in benign neurofibromas.

### Comparison of miRNAs profile between DNFs and PNFs

DNFs and PNFs are both benign nerve stealth tumors but PNFs can undergo malignant transformation, in contrast to DNFs. Hence, we first compared miRNAs expression between DNFs and PNFs. MiRNAs were considered as significantly differentially expressed between DNFs and PNFs when the *P*-value of the non-parametric comparison Mann–Whitney test was less than 0.05 (*P* < 0.05). Eleven miRNAs met these criteria and were considered as displaying a markedly different expression between DNFs and PNFs (Table 1). Among these 11 miRNAs, ten were upregulated (fold changes ranged from 1.76 to 8.57) and only one (miR-203) was downregulated (fold change = 0.13) in PNFs as compared to DNFs. The only downregulated miRNA in PNFs compared to DNFs (miR-203) has previously been described to be silenced in various types of malignancies.

**Table 1:** List of the significantly deregulated miRNAs in plexiform neurofibromas relative to dermal neurofibromas

**miRNAs**	**Functionally verified gene target(s)**	**Location**	**Host gene**	**Dermal neurofibromas (n = 9)**	**Plexiform neurofibromas (n = 41)**	**Fold change**^**a**^	***P***^***b***^	**Peripheral nerves (n = 2)**^**c**^
**Significantly upregulated miRNAs in plexiform neurofibromas relative to dermal neurofibromas**								
miR-486-3p	*PTEN*[10]	8p11.2	*ANK1*	1.3 [0.03-12.8]^d^	9.3 [0.02-198.0]	7.41	0.002	1.4 [0.90-1.8]
miR-185		22q11.2		2.8 [0.10-23.6]	16.0 [0.04-97.5]	5.67	0.003	8.4 [3.1-13.8]
miR-362-5p		Xp11.2	*CLCN5*	4.8 [0.09-72.6]	18.5 [0.03-312.0]	3.90	0.008	21.3 [7.2-35.4]
miR-370	*MAP3K8*[11]	14q32.2		13.8 [5.4-33.0]	28.7 [0.08-232.6]	2.08	0.008	11.5 [10.8-12.1]
miR-491-5p		9p21.3		12.8 [2.0-22.1]	22.6 [0.02-115.3]	1.76	0.014	27.6 [23.8-31.3]
miR-143	*KRAS*[12]	5q32		185.0 [68.1-411.0]	437.7 [3.3-3156.7]	2.37	0.016	426.1 [339.9-512.4]
miR-193a-5p		17q11.2		2.0 [0.58-13.5]	16.9 [0.02-168.9]	8.57	0.017	11.3 [8,0-14.5]
miR-483-5p		11p15.5	*IGF2*	7.4 [0.27-24.2]	19.7 [0.02-326.9]	2.67	0.020	112.3 [38.7-185.8]
miR-181a	*KRAS*[13] and *ATM*[14]	1q32.1 and 9q33.3	*NR6A1* (9q33.3)	8.2 [0.71-84.3]	36.3 [0.02-231.0]	4.44	0.022	10.7 [5.7-15.7]
miR-145	*RREB1*[15]	5q32		436.3 [259.8-2572.9]	1792.9 [22.9-14808.8]	4.11	0.024	830.3 [688.1-972.4]
**Significantly down-regulated miRNA in plexiform neurofibromas relative to dermal neurofibromas**								
miR-203		14q32.3		36.2 [1.2-10966.7]	4.8 [0.05-2943.7]	0.13	0.010	2.6 [2.3-2.8]

^a^Median expression in plexiform neurofibromas/Median expression in dermal neurofibromas.

^b^Mann-Whitney’s U Test.

^c^Non tumorigenic controls.

^d^Median [range] of miRNAs levels.

### Comparison of miRNAs profile between PNFs and MPNSTs

One hundred thirteen miRNAs were differentially expressed between PNFs and MPNSTs (*P* < 0.05): 103 were upregulated (Additional file 1: Table S3) in MPNSTs and 10 were downregulated (fold changes ranged from 0.19 to 0.61; Table 2). We observed high statistical significant upregulation (*P* < 0.00001) of 28/103 upregulated miRNAs in MPNSTs compared to PNFs (fold changes ranged from 2.4 to 2616.7; Table 2).

**Table 2:** List of the most significantly deregulated miRNAs in MPNSTs relative to plexiform neurofibromas

**miRNAs**	**Functionally verified target(s)**	**Localization**	**Host gene**	**Plexiform neurofibromas (n = 41)**	**MPNSTs (n = 15)**	**Fold change**^***a***^	***P***^***b***^	**Peripheral nerves (n = 2)**^**c**^
**Significantly down-regulated miRNAs in MPNSTs relative to plexiform neurofibromas (n = 10)**								
miR-139-5p		11q13.4	*PDE2A*	368.8 [5.6-1523.2]^d^	71.8 [27.6-247.8]	0.19	< 0.00001	253.1 [149.9-356.3]
miR-150		19q13.3		1910.1 [4.4-6800.1]	529.6 [105.6-3531.6]	0.28	< 0.0001	1573.4 [1428.9-1717.9]
miR-338-3p		17q25.3	*AATK*	10.7 [0.03-115.4]	2.3 [0.02-29.8]	0.21	< 0.001	125.6 [88.7-162.5]
miR-195	*CCND1*, *CDK6*, *E2F3*[16]*CCNE1*[17] and *RAF1*[18]	17p13.1		1689.4 [0.03-9958.6]	503.8 [166.6-2233.1]	0.30	< 0.001	689.6 [633.5-745.7]
miR-146a		5q34		3117.1 [85.4-49237.4]	1252.7 [186.9-65865.7]	0.40	< 0.001	3705.8 [2932.0-4479.5]
miR-95		4p16.1	*ABLIM2*	44.5 [0.02-175.7]	26.6 [3.6-56.2]	0.60	0.001	12.7 [7.5-18.0]
let-7b	*RAS*[19], *CCND1*[20] and *HMGA2*[21]	22q13.3		1642.3 [15.8-12746.3]	543.6 [63.9-1531.6]	0.33	0.003	2777.1 [1011.0-4543.1]
miR-186		1p13.1	*ZRANB2*	2030.1 [1.6-3193.4]	1240.9 [727.8-7687.8]	0.61	0.003	2303.1 [1717.4-2889.2]
miR-885-5p		3p25.3	*ATP2B2*	52.4 [0.51-393.3]	20.6 [2.4-62.4]	0.39	0.006	67.2 [46.5-87.9]
miR-200c	*ZEB1*[22]*and ZEB2*	12p13.3		8.4 [0.51-861.1]	4.8 [0.01-22.4]	0.57	0.008	8.1 [6.1-10.0]
**Most significantly upregulated miRNAs in MPNSTs relative to plexiform neurofibromas (n = 28)**								
miR-135b	*APC*[23]	1q32.1		0.41 [0.02-12.0]	1060.9 [4.2-3562.1]	2616.7	< 10^-6^	3.4 [0.57-6.3]
miR-449a	*CCND1* and *HDAC1*[24]	5q11.2	*CDC20B*	0.45 [0.00-11.4]	14.4 [0.28-25.9]	32.30	< 10^-6^	4.0 [3.4-4.6]
miR-210	*HOXA1*, *HOXA9*, *HOXA3*, and *E2F3*[25]	11p15.5		9.8 [0.05-254.1]	236.0 [3.2-458.5]	24.00	< 10^-6^	69.2 [19.0-119.4]
miR-301b	*PTEN*[26]	22q11.2		0.81 [0.02-8.2]	17.3 [1.9-121.6]	21.19	< 10^-6^	0.20 [0.03-0.37]
miR-301a		17q22	*SKA2*	8.7 [0.02-87.8]	173.7 [23.0-592.2]	20.04	< 10^-6^	3.7 [3.3-4.0]
miR-9	*CDH1*[27] and *CDX2*[28]	1q22, 5q14.3 and 15q26.1		52.9 [0.03-472.9]	787.6 [2.9-1265.6]	14.90	< 10^-6^	411.7 [400.8-422.5]
miR-130b		22q11.2		8.3 [0.02-53.5]	66.2 [23.3-442.7]	7.96	< 10^-6^	7.0 [5.5-8.6]
miR-454		17q22	*SKA2*	257.3 [0.05-631.7]	1259.4 [494.9-2684.4]	4.90	< 10^-6^	508.4 [481.2-535.7]
miR-19a	*PTEN*[29] and *CCND1*[30]	13q31.3		201.3 [0.04-764.3]	749.1 [429.9-2078.0]	3.72	< 10^-6^	146.9 [141.3-152.5]
miR-106b	*PTEN, CCND1*, and *E2F1*[31,32]	7q22.1	*MCM7*	126.5 [0.05-483.2]	439.2 [233.9-1795.6]	3.47	< 10^-6^	81.9 [80.1-83.8]
miR-135a	*APC*[23]	12q23.1 and 3p21.1	*GLYCTK* (3p21.1)	0.05 [0.01-4.4]	25.7 [0.01-158.3]	537.54	< 0.00001	65.8 [0.02-131.5]
miR-137		1p21.3		0.08 [0.01-40.2]	21.4 [0.07-82.0]	258.68	< 0.00001	17.6 [3.1-32.1]
miR-31		9p21.3		6.4 [0.02-2747.4]	1581.9 [0.02-8261.8]	246.61	< 0.00001	83.0 [13.7-152.4]
miR-129-3p	*CDK6*	7q32.1 and 11p11.2		0.04 [0.00-2.5]	3.8 [0.01-1409.7]	92.36	< 0.00001	0.94 [0.48-1.4]
miR-224		Xq28	*GABRE*	7.0 [0.04-135.0]	120.2 [8.0-748.0]	17.26	< 0.00001	14.7 [9.6-19.9]
miR-10b	*NF1*, *HOXA3* and *HOXD10*[33,34]	2q31.1		49.1 [0.02-832.3]	649.6 [12.4-1640.1]	14.19	< 0.00001	64.8 [11.7-117.9]
miR-148a		7p15.2		50.2 [0.04-450.0]	413.6 [3.8-793.2]	8.24	< 0.00001	8.5 [5.2-11.9]
miR-18a		13q31.3		3.0 [0.02-38.4]	21.2 [6.4-104.6]	7.02	< 0.00001	5.9 [1.4-10.4]
miR-452		Xq28	*GABRE*	9.9 [0.18-82.0]	58.6 [14.6-260.7]	5.93	< 0.00001	19.5 [19.1-19.9]
miR-598		8p23.1	*XKR6*	15.8 [0.04-105.7]	93.3 [5.0-524.7]	5.91	< 0.00001	29.9 [29.0-30.8]
miR-196b	*HOXB8*[34]	7p15.2		29.7 [0.54-526.4]	165.2 [12.5-788.5]	5.56	< 0.00001	124.9 [70.5-179.3]
miR-425		3p21.3	*DALRD3*	16.3 [0.11-116.5]	84.4 [38.3-105.1]	5.18	< 0.00001	19.2 [11.5-27.0]
miR-10a	*HOXD10*[35]	17q21.3	*HOXB3*	53.1 [0.04-498.1]	230.1 [67.6-400.7]	4.33	< 0.00001	21.3 [14.2-28.3]
miR-93		7q22.1	*MCM7*	149.2 [0.04-860.2]	607.8 [303.6-2824.3]	4.07	< 0.00001	142.8 [114.4-171.2]
miR-20a	*CCND1* and *E2F1*[31]	13q31.3		295.6 [0.02-1269.4]	1064.2 [196.3-3498.9]	3.60	< 0.00001	531.2 [390.0-672.5]
miR-19b		13q31.3 and Xq26.2		1757.9 [0.99-5939.3]	4761.9 [2678.3-11811.4]	2.71	< 0.00001	3039.0 [2435.6-3642.5]
miR-484		Xq26.2		1354.6 [70.2-4301.5]	3537.7 [1213.0-8554.1]	2.61	< 0.00001	1047.4 [438.8-1656.1]
miR-192		11q13.1		38.8 [0.02-95.4]	94.3 [39.7-236.6]	2.43	< 0.00001	27.4 [18.7-36.1]

^a^Median expression in MPNSTs/Median expression in plexiform neurofibromas.

^b^Mann- Whitney’s U Test.

^c^Non tumorigenic controls.

^d^Median [range] of miRNAs levels.

### Hierarchical clustering

**Figure 1:**
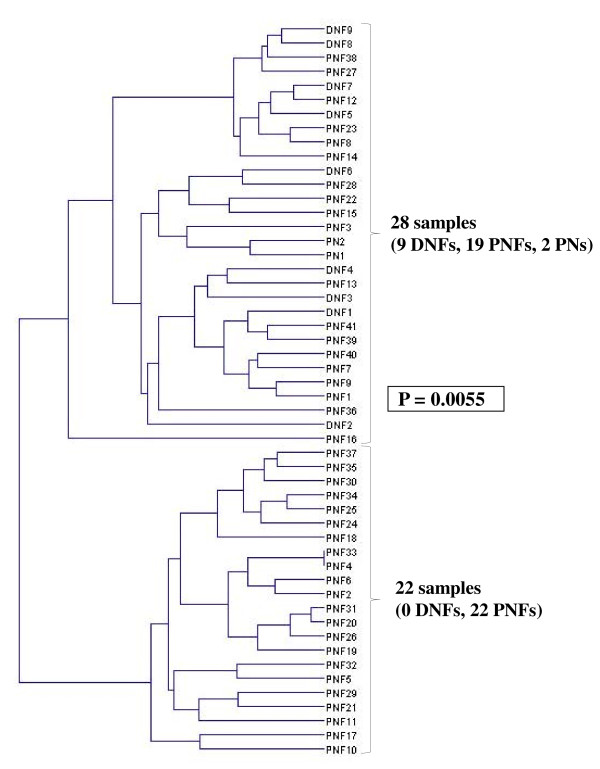
Dendrogram of the nine DNFs, 41 PNFs, and two peripheral nerves (PN1 and PN2) constructed using a supervised hierarchical clustering with UPGMA algorithm according to the expression of the 11 miRNAs significantly deregulated between DNFs and PNFs.

**Figure 2:**
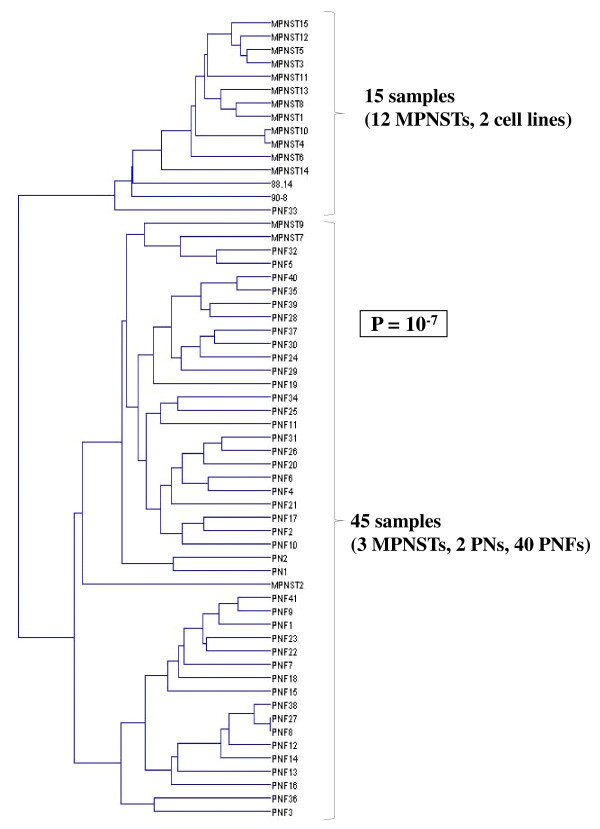
Dendrogram of the 41 PNFs, 15 MPNSTs, two peripheral nerves and two MPNST cell lines constructed using a supervised hierarchical clustering with UPGMA algorithm according to the expression of the 28 miRNAs most significantly deregulated between PNFs and MPNSTs.

To identify a miRNA signature that could be a useful adjunct to NF1-associated tumor diagnosis, a hierarchical clustering was performed. Hierarchical clustering of the nine DNF and 41 PNF samples, based on the expression of the 11 significantly deregulated miRNAs between those two types of tumors (Table 1) identified two main groups of tumor samples with exclusively PNFs in one group (*P* = 0.0055; Figure 1). Both peripheral nerve samples (PN1 and PN2) clustered in the DNFs-containing subgroup. We also selected the 38 most strongly deregulated miRNAs between PNFs and MPNSTs (Table 2) to perform a hierarchical clustering of these two types of tumors. Analysis of the dendrogram based on the expression of those 38 miRNAs allowed us to identified two main groups, with 98% of PNFs (40/41) clustered in one group and 80% of MPNSTs (12/15) in the other group (*P* < 10^-7^; Figure 2). Interestingly, MPNSTs cell lines clustered with the MPNSTs-containing subgroup.

### Coregulation of physically clustered miRNAs

We hypothesized that clustered miRNAs (i.e. located at the same locus) may function as a single transcription unit. Among upregulated miRNAs in MPNSTs compared to PNFs (Table 2; Additional file 1: Table S3), we identified several clustered miRNAs: miR-301b and miR-130b are located in 22q11.2, miR-301a and miR-454 are located in 17q22 in the first intron of the *SKA2* gene, miR-224 and miR-452 are located in Xq28 inside the *GABRE* gene, miR-106b and miR-93 are located in 7q22 within intron 13 of the *MCM7* gene, and miR-19a, miR-18a, miR-20a, and miR-19b belong to the polycistronic cluster miR-17 ~ 92 located in 13q31.3. Similarly, among the miRNAs upregulated in PNFs compared to DNFs (Table 1), miR-143 and miR-145 belong to the same cluster located in 5q32. A Spearman’s rank correlation test demonstrated that miR-301b and miR-130b (r = +0.67, *P* = 3.1.10^-6^), miR-301a and miR-454 (r = +0.48, *P* = 1.7.10^-3^), miR-224 and miR-452 (r = +0.77, *P* = 7.6.10^-8^), miR-106b and miR-93 (r = +0.84, *P* < 10^-8^), and miR-143 and miR-145 (r = +0.90, *P* < 10^-8^) have positive correlated expression. In addition, we showed that both miR-224 (Spearman’s rank correlation coefficient = +0.49, *P* = 0.002) and miR-452 (r = +0.47, *P* = 0.002) have positive correlated expression with their host gene, *GABRE*.

### Coregulation of paralogous miRNAs

We hypothesized that paralogous miRNAs may target the same protein-coding genes, acting as synergistic co-regulators of shared target genes. Among the most significantly upregulated miRNAs in MPNSTs compared to PNFs (Table 2), two couples of paralogous miRNAs differ in only few base pairs: miR-301a and miR-301b (targeting *NF1*), and miR-10a and miR-10b (targeting *PTEN*). Using a Spearman test, we demonstrated that expression of miR-301a and miR-301b (r = +0.65, *P* = 6.7.10^-6^), and miR-10a and miR-10b (r = +0.75, *P* = 1.5.10^-7^) have significantly positive correlated expression. These paralogous miRNAs have high sequence identity but are located at different *loci* and chromosomes. Their correlated overexpressions in MPNSTs may depend on common regulation and could reflect a cooperative activity to switch off shared target genes.

### Inverse expression of miRNAs with their protein-coding gene targets

Using Spearman’s rank correlation test, we reported that several upregulated miRNAs in MPNSTs compared to PNFs have significant inverse correlated expression with their previously identified target genes: *HMGA2* mRNA expression is inversely correlated with let-7b (r = −0.387, *P* = 0.003) and *PTEN* expression is inversely correlated with miR-301a (r = −0.67, *P* = 5.9.10^-8^), miR-19a (r = −0.67, *P* = 6.4.10^-8^), and miR-106b (r = −0.65, *P* = 1.5.10^-7^).

### Expression of four miRNAs processing machinery components: *DICER*, *DROSHA*, *DGCR8* and *AGO2*

Alterations of miRNAs processing machinery components expression have been reported in human tumors [36]. We therefore explored the possibility that global miRNA dysregulation observed in NF1 tumorigenesis could be due to altered expression of *DICER*, *DROSHA*, *DGCR8* and/or *AGO2*, and their expressions were determined. No significant differences in *DICER*, *DROSHA*, *DGCR8* and *AGO2* mRNA levels were found in PNFs compared to DNFs or in MPNSTs compared to PNFs (*P* > 0.01; Additional file 1: Table S4). These observations reinforce the specificity of the identified signature (11 deregulated miRNAs in PNFs and 113 in MPNSTs) *versus* a global miRNAome deregulation caused by a tumor alteration of miRNAs biogenesis.

## Discussion

Recent evidences indicate that miRNA network play a critical role in the regulation of gene expression involved in tumor development and progression. In this study, we applied a RT-PCR analysis to comprehensively characterize the expression pattern of 377 miRNAs in a large series of benign and malignant NF1-related nerve sheath tumors.

Eleven miRNAs were found to be differentially expressed between DNFs and PNFs (Table 1). Interestingly, the most significantly upregulated miRNA in PNFs, miR-486-3p, targets the major tumor suppressor gene, *PTEN*[10]. *PTEN* expression is frequently decreased in a wide spectrum of human cancers with several miRNAs being validated as *PTEN* regulators [37]. Using murine conditional deletion of *Pten* and activation of *Kras*, Gregorian *at al.* suggested that *PTEN* dosage is critical for formation of PNFs [8]. MiRNAs regulation typically allows such subtle modulations in gene expression dosage. It is important to note that the two tested peripheral nerve samples (non-tumorigenic samples) showed similar level expression of miR-486-3p than DNF samples (Table 1). Overexpression of miR-486-3p in PNFs may therefore specifically reflect an abnormal upregulation in these tumors. We further hypothesised that miR-486-3p may be a major onco-miR by downregulating *PTEN* in PNFs development.

Our results also reveal overexpression of miR181a in PNFs. MiR181a targets *ATM*[14], a tumor suppressor that regulates the p53 pathway [38] that has been shown to be essential for PNFs formation in transgenic mouse models [7]. Similar level expression of miR181a was found in both peripheral nerve samples and in DNFs samples (Table 1).

Finally, three other markedly overexpressed miRNAs in PNFs compared to DNFs have previously been demonstrated to negatively regulate the RAS signaling pathway: miR-370 targets *MAP3K8*[11], miR-143 targets *KRAS*[12,13], and miR-145 targets *RREB1*[15]. Biallelic loss of function of the *NF1* gene in neurofibromas promotes activation of the RAS signaling pathway. We assume that overexpression of these four miRNAs may reveal a feedback loop that attempts to correct hyperactivation of RAS signaling. Interestingly, miR-143 and miR-145 are both located at 5q32 and have positive correlated expressions, suggesting a common transcription unit, allowing a concerted action on RAS pathway.

We also investigated the 113 differentially expressed miRNAs between PNFs and MPNSTs. Among the 103 upregulated and the ten downregulated miRNAs in MPNSTs, we further analyzed the most markedly differentially expressed miRNAs (Table 2). A subset of miRNAs (miR-301a [26], miR19a [29], and miR-106b [31,32]) previously demonstrated to directly target the suppressor *PTEN* was found to be overexpressed in MPNSTs. Recent transgenic mice model with *Pten* and *Nf1* conditional knock out in Schwann cells, implicated the synergistic role of *Pten* and *Nf1* inactivation in MPNSTs development [23]. We confirmed that *PTEN* expression was inversely correlated with miR-301a, miR-19a, and miR-106b. Interestingly, we showed that paralogs miR-301a and miR-301b have significantly positive correlated expression suggesting that these two miRNAs may synergistically target *PTEN*.

We also identified a panel of five deregulated miRNAs (miR-135a, miR-135b, miR-9, miR-200c, and let-7b) that are major regulators of epithelial-mesenchymal transition (EMT). MiR-135b and miR-135a represent the most overexpressed miRNAs in MPNSTs compared to PNFs (Table 2). Members of the miR-135 family target the *t*umor suppressor *APC*, a key negative regulator of the WNT canonical pathway involved in tumorigenesis [39]. E-cadherin [27] is a major epithelial *adherens* junction protein which plays a critical role in EMT. MiR-9 is also overexpressed in MPNSTs and it has been previously demonstrated that miR-9 is upregulated in breast cancer cells and targets the *E-cadherin* gene (also named *CDH1*) [40].

MiR-200c and let-7b are both downregulated in MPNSTs compared to PNFs. Members of miR-200 family act as critical regulators of EMT and contribute to the acquisition of invasive behavior in many types of cancer [22]. They negatively regulate expression of *ZEB1*[19] and *ZEB2*, two E-box binding transcription factors that are powerful regulators of E-cadherin expression and several other effectors involved in epithelial polarity. Let-7 family miRNAs regulate cell proliferation and differentiation and are frequently downregulated in many human cancers [21]. They are considered as tumor suppressor miRNAs, notably through targeting genes with oncogenic activity such as *RAS*[21] and *HMGA2*[41]. We confirmed that *HMGA2* expression is inversely correlated with let-7b. HMGA2 enhances *TWIST* (whom expression is regulated by WNT signaling) and inhibits *CDKN2A*/*CDKN2B* gene expression, corresponding with aggressive behavior [42-44]*.* Hyperactivation of RAS signaling pathway, overexpression of *TWIST1*[6] and *CDKN2A* inactivation [3] have frequently been reported in MPNSTs. Together, upregulation of miR-135a, miR-135b, and miR-9 and downregulation of miR-200c and let-7b in MPNSTs may thus contribute to mesenchymal transition with acquisition of an invasive behavior in these malignant aggressive tumors.

Our results also independently confirm that miR-10b is significantly upregulated in MPNSTs compared to PNFs. Expression of miR-10b is induced by the metastasis-promoting transcription factor TWIST [44]. Chai *et al.* previously showed overexpression of miR-10b in MPNSTs and demonstrated that miR-10b targets *NF1* mRNA [44]. MiR-10b controls expression of the pro-metastatic RhoC via directly targeting *HOXD10* mRNA [33]. *HOXD10* encodes a homeobox (HOX) protein that act as a transcriptional repressor of *RHOC*. Aberrant expression of RHO GTPases or RHO effectors has been described in various cancer types, suggesting the implication of RHO signaling pathway in NF1 tumorigenesis [34]. Interestingly, our analysis of miRNAs expression in MPNSTs revealed a significant upregulation of others miRNAs that target *HOX* genes (Table 2): miR-196b targets *HOXB8*[35], miR-210 targets *HOXA1*, *HOXA9*, and *HOXA3*[25], miR-10a and miR-10b target *HOXA3* and *HOXD10*[28], and miR-9 target *CDX2*[45]. *HOX* genes represent a large group of related genes encoding transcription factors with crucial roles in development, apoptosis, differentiation, and cell motility [46].

Among the most markedly differentially expressed miRNAs in MPNSTs (Table 2), we also identified a panel of significantly deregulated miRNAs (including miR-195, let-7b, miR-210, miR-20a, miR-19a, miR-449a, miR-129-3p, and miR-106b) that target major regulators of cell cycle. MiR-195 is downregulated in MPNSTs and has recently been described as a tumor suppressor in various types of cancers [16]. MiR-195 targets several G1/S transition-related genes such as cyclin D1 (*CCND1*), *CDK6*, *E2F3*[17], and *CCNE1*[20]. Let-7b (downregulated in MPNSTs, Table 2) has also been shown to repress *CCND1* expression [30]. In addition miR-210 targets *E2F3*[25], miR-20a targets *CCND1* and *E2F1*[31], miR-19a and miR-449a target *CCND1*[24], miR-129-3p targets *CDK6*[47], and miR-106b targets *CCND1* and *E2F1*[31]*.* Interestingly, *NF1* inactivation has previously been shown to enhance *CCND1* expression in Schwann cells [18]. Overexpression of *CCND1* could thus account for a major feature of the Schwann cells proliferation in MPNSTs formation. Moreover, miR-210 has been described as a key regulator of the hypoxia response in tumor onset identified as a major *HIF1A*-induced miRNA, and makes link between hypoxia and the regulation of cell cycle [25]. We confirmed that miR-210 has a significant positive correlated expression with *HIF1A* (r = +0.57; *P* = 5.8.10^-6^).

A subset of significantly deregulated miRNAs in MPNSTs (let-7b, miR-195, and miR-10b) has previously been described to target members of the RAS-MAPK pathway. Let-7b is a member of the let-7 miRNAs family which target RAS [21]. Recent data report that miR-195 targets RAF1 [48], a direct downstream effector of RAS. Interestingly, let-7b and miR-195 are both downregulated in MPNSTs compared to PNFs and could subsequently act as indirect activator of the RAS-MAPK pathway. Additionally, miR-10b is upregulated in MPNSTs and has been previously demonstrated to target *NF1*[44].

## Conclusion

In conclusion, miRNAome profiling of NF1-associated peripheral nerve sheath tumors suggests intricately deregulated associated molecular networks. We identified several subsets of markedly deregulated miRNAs in PNFs, in particular miR-486-3p that targets *PTEN*, a major component of the RAS-PI3K-AKT signaling pathway. Our work highlights the key role of miRNAs in NF1-associated tumorigenesis, leading to subtle reductions in *PTEN* levels. Our result also confirmed the major role of *PTEN* in MPNSTs formation. Our study identifies miRNAs clearly deregulated in MPNSTs and involved in oncogenic events including loss of cell adhesion and metastatic behavior mediated by EMT, deregulation of *HOX* genes expression, increased cell cycle progression, and RAS-MAPK aberrant signaling. All of these miRNAs should be further investigated using *in vitro* or *in vivo* experiments to fully understand their role in the formation of neurofibromas and MPNSTs and to identify mechanisms involved in their deregulation. Finally, our results may serve for the basis of potential diagnostic and predictive biomarkers and represent novel strategies for effective pharmacological therapies of NF1 tumors.

## Methods

### Patients and samples

Tissue samples of nine DNFs, 41 PNFs, and 15 MPNSTs were obtained after surgical excision from NF1 patients followed in the Neurofibromatosis National Reference Center (Henri Mondor Hospital, Créteil, France). Tumors classification was confirmed at the histological level by our NF1 reference pathologist (N.O.). Main characteristics of the 15 studied patients with MPNST are presented in Additional file 1: Table S1. Immediately after excision, tumor samples were frozen in liquid nitrogen and stored at −80°C. All of the patients fulfilled the national institute of health (NIH) diagnostic criteria for NF1. Adult sciatic and tibial nerves from two different control subjects with no NF1 were also analyzed (Department of Orthopedic Surgery, Cochin Hospital, Paris, France). The study was approved by the local ethics committee and all the participants gave their written informed consent. Two MPNST cell lines established from NF1 patients (ST88-14 and 90–8) were kind gifts from Pr. Nancy Ratner (Cincinnati Children's Hospital Medical Center, USA).

### RNA extraction

Total RNA was extracted from frozen tumor samples, MPNSTs cell lines, and fresh adult nerves by using the acid-phenol guanidinium method. The quantity of the RNA samples was assessed with a Nanodrop spectrophotometer (Coleman Technologies, Orlando, FL). The quality of the RNA samples was determined by electrophoresis through agarose gels and staining with ethidium bromide.

### MiRNAs expression analysis

Reverse transcription reaction was performed using the TaqMan MicroRNA Reverse Transcription Kit (Applied Biosystems, Foster City, CA) according to the manufacturer’s instructions. Taqman primers for the miRNA arrays (Megaplex RT Human Pool A) were used for reverse transcription and thermal cycling was performed over 40 cycles (16°C for 2 min, 42°C for 1 min, 50°C for 1 s), after which the reactions were held at 85°C for 5 min and then at 4°C. Expression of 377 miRNAs from the Sanger database v12 (Additional file 1: Table S2) was subsequently analyzed in all the samples using the 384-wells microfluidic TaqMan Low Density Array (Applied Biosystems TaqMan human microRNA cardA v2.0 composed of 384 single assays including 377 highly characterized human miRNAs) based on stem-loop real-time PCR based TaqMan miRNA expression assay. Each sample was normalized on the basis of its miR-191 content. We selected miR-191 as endogenous control because this miRNA is known to be one of the most stably expressed miRNAs across different normal and tumor tissues [49]. Cycle threshold (Ct) values were obtained using the SDS software v2.3 set with automaticbaseline. Each result was determined as a difference in target miRNA expression relative to miR-191 and expressed as 2^ΔCt^. This value of the samples was subsequently normalized such that a sample with a Ct of 32, considered as the limit of quantification of the system, showed a value of 1.

### Protein-coding genes real-time RT-PCR

We quantified the mRNA level of the following genes: four genes encoding components of the miRNAs biogenesis pathway (*DICER*, *DROSHA*, *DGCR8* and *AGO2*), three miRNAs host genes (*SKA2*, *GABRE*, and *MCM7*), two miRNAs target genes (*PTEN* and *HMGA2*), and one known inducer of miR-210 (*HIF1A*). The practical aspects of real-time quantitative RT-PCR using the ABI Prism 7900 Sequence Detection System (Applied Biosystems) have been described in detail elsewhere [50]. Briefly, each sample expression was normalized on the basis of its content of an endogenous control gene (*TBP*, *TATA box binding protein*) and such that the median expression in the DNFs values was 1. The nucleotide sequences of the oligonucleotide primers used to amplify the different target genes are available on demand.

### Clustering and statistical analysis

As miRNA expression levels did not fit a Gaussian distribution, (i) miRNA levels in each group of tumors were expressed as their median values rather than their mean values and (ii) comparisons of each miRNA between tumors groups were tested by using the non parametric Mann–Whitney *U* test. Clustering was performed using the UPGMA (Unweighted Pair Group Method with Arithmetic Mean) hierarchical algorithm with GeneANOVA software. We performed a chi-square test to statistically compare the tumors distribution in hierarchical clustering. We applied Spearman correlation test to identify pairs of miRNAs with similar expression patterns and correlated expression between miRNAs and protein-coding genes.

### Availability of supporting data

The data set supporting the results of this article are included within the article and its Additional file 1.

## Competing interests

The authors declare that they have no competing interests.

## Authors’ contributions

MV, DV, IB, and PW participated in the design of the study, and interpretation of data. KL participated in tumor samples collection. NO performed tumors anatomopathologic analysis. LVA, and PW provided clinical follow-up information of the patient cohort. MH, VD, and LL performed tumor surgical excision. JV, IL, and AL performed the experiments and JMP and IB were responsible for data analysis. IB, JMP, and EP have been involved in writing the manuscript with assistance from BP, and MH. All authors read and approved the final manuscript.

## Supplementary Material

Additional file 1: Table S1 Clinical and histological characteristics of the 15 patients with MPNST. **Table S2** Complete list of the 377 miRNAs analyzed from the TaqMan human microRNA cardA v2.0 (Applied Biosystems). **Table S3** Complete list of the 103 significantly upregulated genes in MPNSTs relative to plexiform neurofibromas. **Table S4** MRNA levels of four miRNAs processing machinery component genes (*DICER*, *DROSHA*, *DGCR8* and *AGO2*32) in nine dermal neurofibromas, 41 plexiform neurofibromas, and 15 MPNSTs. Click here for file
