# Supplementary material for: MicroRNAome profiling in benign and malignant neurofibromatosis type 1-associated nerve sheath tumors: evidences of PTEN pathway alterations in early NF1 tumorigenesis
Source: BMC Genomics. 2013 Jul 13;14:473. doi: 10.1186/1471-2164-14-473 (PMC3744175; doi:10.1186/1471-2164-14-473)
Supplement: Additional file 1: Table S1 — Clinical and histological characteristics of the 15 patients with MPNST. Table S2 Complete list of the 377 miRNAs analyzed from the TaqMan human microRNA cardA v2.0 (Applied Biosystems). Table S3 Complete list of the 103 significantly upregulated genes in MPNSTs relative to plexiform neurofibromas. Table S4 MRNA levels of four miRNAs processing machinery component genes (DICER, DROSHA, DGCR8 and AGO232) in nine dermal neurofibromas, 41 plexiform neurofibromas, and 15 MPNSTs. [file 1471-2164-14-473-S1.doc]

**Additional file 1: Table S1.** Clinical and histological characteristics of the 15 patients with MPNST.

| **Patient**  **Tumor/Sex/Age (years)** | **Pain** | **Enlargement**  **of mass** | **Neurological**  **signs** | **Tumor**  **localization** | **Tumor**  **size (cm)** | **Histoprognostic**  **grade** |
| --- | --- | --- | --- | --- | --- | --- |
| M1/F/19 | + | + | - | Lower limb | 27 | III |
| M2/F/18 | + | - | - | Upper limb | 20 | III |
| M3/M/18 | - | + | - | Head | 15 | III |
| M4/M/35 | + | + | + | Abdomen | 20 | III |
| M5/M/34 | + | + | + | Lumbar | 6.5 | III |
| M6/M/53 | + | + | + | Upper limb | 13 | III |
| M7/F/24 | + | + | + | Lumbar | 7 | I |
| M8/M/24 | + | + | + | Abdomen | 20 | III |
| M9/M/31 | - | + | - | Face | 15 | III |
| M10/F/29 | + | + | - | Trunk | 10 | III |
| M11/M/42 | + | + | - | Abdomen | 25 | III |
| M12/M/42 | + | + | - | Abdomen | 25 | III |
| M13/F/25 | + | + | - | Buttock | 20 | III |
| M14/M/29 | + | + | - | Lower limb | 20 | III |
| M15/F/37 | + | + | - | Upper limb | 20 | III |

Plus sign indicates present; minus sign, absent; F, Female; M, male

**Additional file 1: Table S2.** Complete list of the 377 miRNAs analyzed from the TaqMan human microRNA cardA v2.0 (Applied Biosystems).

| let-7a | miR-15b | miR-23a | miR-372 | miR-502-5p | miR-548d-3p | miR-95 |
| --- | --- | --- | --- | --- | --- | --- |
| let-7b | miR-16 | miR-23b | miR-373 | miR-503 | miR-548d-5p | miR-96 |
| let-7c | miR-17 | miR-24 | miR-374a | miR-504 | miR-551b | miR-98 |
| let-7d | miR-181a | miR-25 | miR-374b | miR-505 | miR-556-3p | miR-99a |
| let-7e | miR-181c | miR-26a | miR-375 | miR-506 | miR-556-5p | miR-99b |
| let-7f | miR-182 | miR-26b | miR-376a | miR-507 | miR-561 |  |
| let-7g | miR-183 | miR-27a | miR-376b | miR-508-3p | miR-570 |  |
| miR-1 | miR-184 | miR-27b | miR-376c | miR-508-5p | miR-574-3p |  |
| miR-100 | miR-185 | miR-28-3p | miR-377 | miR-509-3p | miR-576-3p |  |
| miR-101 | miR-186 | miR-28-5p | miR-379 | miR-509-5p | miR-576-5p |  |
| miR-103 | miR-187 | miR-296-3p | miR-380 | miR-510 | miR-579 |  |
| miR-105 | miR-188-3p | miR-296-5p | miR-381 | miR-511 | miR-582-3p |  |
| miR-106a | miR-18a | miR-298 | miR-382 | miR-512-3p | miR-582-5p |  |
| miR-106b | miR-18b | miR-299-3p | miR-383 | miR-512-5p | miR-589 |  |
| miR-107 | miR-190 | miR-299-5p | miR-384 | miR-513-5p | miR-590-5p |  |
| miR-10a | miR-191 | miR-29a | miR-409-5p | miR-515-3p | miR-597 |  |
| miR-10b | miR-192 | miR-29b | miR-410 | miR-515-5p | miR-598 |  |
| miR-122 | miR-193a-3p | miR-29c | miR-411 | miR-516a-5p | miR-615-3p |  |
| miR-124 | miR-193a-5p | miR-301a | miR-412 | miR-516b | miR-615-5p |  |
| miR-125a-3p | miR-193b | miR-301b | miR-422a | miR-517a | miR-616 |  |
| miR-125a-5p | miR-194 | miR-302a | miR-423-5p | miR-517b | miR-618 |  |
| miR-125b | miR-195 | miR-302b | miR-424 | miR-517c | miR-624 |  |
| miR-126 | miR-196b | miR-302c | miR-425 | miR-518a-3p | miR-625 |  |
| miR-127-3p | miR-197 | miR-30b | miR-429 | miR-518a-5p | miR-627 |  |
| miR-127-5p | miR-198 | miR-30c | miR-431 | miR-518b | miR-628-5p |  |
| miR-128 | miR-199a-3p | miR-31 | miR-433 | miR-518c | miR-629 |  |
| miR-129-3p | miR-199a-5p | miR-32 | miR-448 | miR-518d-3p | miR-636 |  |
| miR-129-5p | miR-199b-5p | miR-320 | miR-449a | miR-518d-5p | miR-642 |  |
| miR-130a | miR-19a | miR-323-3p | miR-449b | miR-518e | miR-651 |  |
| miR-130b | miR-19b | miR-324-3p | miR-450a | miR-518f | miR-652 |  |
| miR-132 | miR-200a | miR-324-5p | miR-450b-3p | miR-519a | miR-653 |  |
| miR-133a | miR-200b | miR-325 | miR-450b-5p | miR-519c-3p | miR-654-3p |  |
| miR-133b | miR-200c | miR-326 | miR-451 | miR-519d | miR-654-5p |  |
| miR-134 | miR-202 | miR-328 | miR-452 | miR-519e | miR-655 |  |
| miR-135a | miR-203 | miR-329 | miR-453 | miR-520a-3p | miR-660 |  |
| miR-135b | miR-204 | miR-330-3p | miR-454 | miR-520a-5p | miR-671-3p |  |
| miR-136 | miR-205 | miR-330-5p | miR-455-3p | miR-520b | miR-672 |  |
| miR-137 | miR-208 | miR-331-3p | miR-455-5p | miR-520d-5p | miR-674 |  |
| miR-138 | miR-208b | miR-331-5p | miR-483-5p | miR-520e | miR-708 |  |
| miR-139-3p | miR-20a | miR-335 | miR-484 | miR-520f | miR-744 |  |
| miR-139-5p | miR-20b | miR-337-5p | miR-485-3p | miR-520g | miR-758 |  |
| miR-140-3p | miR-21 | miR-338-3p | miR-485-5p | miR-521 | miR-871 |  |
| miR-140-5p | miR-210 | miR-339-3p | miR-486-3p | miR-522 | miR-872 |  |
| miR-141 | miR-211 | miR-339-5p | miR-486-5p | miR-523 | miR-873 |  |
| miR-142-3p | miR-212 | miR-33b | miR-487a | miR-524-5p | miR-874 |  |
| miR-142-5p | miR-214 | miR-340 | miR-487b | miR-525-3p | miR-875-3p |  |
| miR-143 | miR-215 | miR-342-3p | miR-488 | miR-525-5p | miR-876-3p |  |
| miR-145 | miR-216a | miR-342-5p | miR-489 | miR-526b | miR-876-5p |  |
| miR-146a | miR-216b | miR-345 | miR-490-3p | miR-532-3p | miR-885-3p |  |
| miR-146b-3p | miR-217 | miR-346 | miR-491-3p | miR-532-5p | miR-885-5p |  |
| miR-146b-5p | miR-218 | miR-34a | miR-491-5p | miR-539 | miR-886-3p |  |
| miR-147 | miR-219-1-3p | miR-34c-5p | miR-492 | miR-541 | miR-886-5p |  |
| miR-147b | miR-219-2-3p | miR-361-5p | miR-493 | miR-542-3p | miR-887 |  |
| miR-148a | miR-219-5p | miR-362-3p | miR-494 | miR-542-5p | miR-888 |  |
| miR-148b | miR-22 | miR-362-5p | miR-495 | miR-544 | miR-889 |  |
| miR-149 | miR-220 | miR-363 | miR-496 | miR-545 | miR-890 |  |
| miR-150 | miR-220b | miR-365 | miR-499-3p | miR-548a-3p | miR-891a |  |
| miR-152 | miR-220c | miR-367 | miR-499-5p | miR-548a-5p | miR-891b |  |
| miR-153 | miR-221 | miR-369-3p | miR-500 | miR-548b-3p | miR-892a |  |
| miR-154 | miR-222 | miR-369-5p | miR-501-3p | miR-548b-5p | miR-9 |  |
| miR-155 | miR-223 | miR-370 | miR-501-5p | miR-548c-3p | miR-92a |  |
| miR-15a | miR-224 | miR-371-3p | miR-502-3p | miR-548c-5p | miR-93 |  |

**Additional file 1: Table S3.** Complete list of the 103 significantly upregulated genes in MPNSTs relative to plexiform neurofibromas.

| **miRNAs** | **Plexiform neurofibromas (n=41)** | **MPNSTs**  **(n=15)** | **Fold change*^a^*** | ***P^b^*** |
| --- | --- | --- | --- | --- |
| miR-135b | **0.41** [0.02-12.0]^c^ | **1060.9** [4.2-3562.1] | 2616.67 | < 10^-6^ |
| miR-449a | **0.45** [0.00-11.4] | **14.4** [0.28-25.9] | 32.30 | < 10^-6^ |
| miR-210 | **9.8** [0.05-254.1] | **236.0** [3.2-458.5] | 24.00 | < 10^-6^ |
| miR-301b | **0.81** [0.02-8.2] | **17.3** [1.9-121.6] | 21.19 | < 10^-6^ |
| miR-301a | **8.7** [0.02-87.8] | **173.7** [23.0-592.2] | 20.04 | < 10^-6^ |
| miR-9 | **52.9** [0.03-472.9] | **787.6** [2.9-1265.6] | 14.90 | < 10^-6^ |
| miR-130b | **8.3** [0.02-53.5] | **66.2** [23.3-442.7] | 7.96 | < 10^-6^ |
| miR-454 | **257.3** [0.05-631.7] | **1259.4** [494.9-2684.4] | 4.90 | < 10^-6^ |
| miR-19a | **201.3** [0.04-764.3] | **749.1** [429.9-2078.0] | 3.72 | < 10^-6^ |
| miR-106b | **126.5** [0.05-483.2] | **439.2** [233.9-1795.6] | 3.47 | < 10^-6^ |
| miR-135a | **0.05** [0.01-4.4] | **25.7** [0.01-158.3] | 537.54 | < 0.00001 |
| miR-137 | **0.08** [0.01-40.2] | **21.4** [0.07-82.0] | 258.68 | < 0.00001 |
| miR-31 | **6.4** [0.02-2747.4] | **1581.9** [0.02-8261.8] | 246.61 | < 0.00001 |
| miR-129-3p | **0.04** [0.00-2.5] | **3.8** [0.01-1409.7] | 92.36 | < 0.00001 |
| miR-224 | **7.0** [0.04-135.0] | **120.2** [8.0-748.0] | 17.26 | < 0.00001 |
| miR-10b | **49.1** [0.02-832.3] | **649.6** [12.4-1640.1] | 14.19 | < 0.00001 |
| miR-148a | **50.2** [0.04-450.0] | **413.6** [3.8-793.2] | 8.24 | < 0.00001 |
| miR-18a | **3.0** [0.02-38.4] | **21.2** [6.4-104.6] | 7.02 | < 0.00001 |
| miR-452 | **9.9** [0.18-82.0] | **58.6** [14.6-260.7] | 5.93 | < 0.00001 |
| miR-598 | **15.8** [0.04-105.7] | **93.3** [5.0-524.7] | 5.91 | < 0.00001 |
| miR-196b | **29.7** [0.54-526.4] | **165.2** [12.5-788.5] | 5.56 | < 0.00001 |
| miR-425 | **16.3** [0.11-116.5] | **84.4** [38.3-105.1] | 5.18 | < 0.00001 |
| miR-10a | **53.1** [0.04-498.1] | **230.1** [67.6-400.7] | 4.33 | < 0.00001 |
| miR-93 | **149.2** [0.04-860.2] | **607.8** [303.6-2824.3] | 4.07 | < 0.00001 |
| miR-20a | **295.6** [0.02-1269.4] | **1064.2** [196.3-3498.9] | 3.60 | < 0.00001 |
| miR-19b | **1757.9** [0.99-5939.3] | **4761.9** [2678.3-11811.4] | 2.71 | < 0.00001 |
| miR-484 | **1354.6** [70.2-4301.5] | **3537.7** [1213.0-8554.1] | 2.61 | < 0.00001 |
| miR-192 | **38.8** [0.02-95.4] | **94.3** [39.7-236.6] | 2.43 | < 0.00001 |
| miR-431 | **0.13** [0.02-50.4] | **102.8** [0.02-1661.3] | 779.68 | < 0.0001 |
| miR-488 | **0.24** [0.01-7.4] | **8.2** [0.01-41.3] | 33.89 | < 0.0001 |
| miR-221 | **13.9** [0.02-276.9] | **117.4** [3.3-467.3] | 8.42 | < 0.0001 |
| miR-21 | **258.5** [0.02-7652.7] | **1906.4** [331.5-11364.6] | 7.37 | < 0.0001 |
| miR-128 | **3.2** [0.02-47.5] | **21.8** [0.58-48.3] | 6.77 | < 0.0001 |
| miR-376a | **36.6** [0.02-273.9] | **240.9** [3.6-667.1] | 6.58 | < 0.0001 |
| miR-483-5p | **19.7** [0.02-326.9] | **125.1** [3.7-613.9] | 6.36 | < 0.0001 |
| miR-98 | **2.0** [0.02-35.7] | **10.9** [1.7-55.9] | 5.53 | < 0.0001 |
| miR-199a-3p | **2079.3** [244.4-21861.5] | **10570.7** [810.1-37903.5] | 5.08 | < 0.0001 |
| miR-146b-5p | **1237.5** [90.9-7956.5] | **4893.8** [568.2-13792.5] | 3.95 | < 0.0001 |
| miR-324-5p | **11.3** [0.08-122.9] | **44.5** [8.0-190.2] | 3.92 | < 0.0001 |
| miR-181a | **36.3** [0.02-231.0] | **135.6** [26.2-725.8] | 3.74 | < 0.0001 |
| miR-214 | **961.6** [86.2-6799.3] | **3586.1** [142.1-9388.6] | 3.73 | < 0.0001 |
| miR-155 | **94.7** [0.29-309.1] | **264.9** [77.1-1864.4] | 2.80 | < 0.0001 |
| miR-590-5p | **56.1** [0.02-200.3] | **138.3** [12.1-602.7] | 2.46 | < 0.0001 |
| miR-194 | **13.1** [0.03-46.6] | **26.5** [10.4-69.2] | 2.03 | < 0.0001 |
| miR-17 | **2477.0** [2.2-7064.3] | **4975.2** [2038.0-18880.0] | 2.01 | < 0.0001 |
| miR-106a | **3021.7** [2.0-8379.3] | **5163.2** [2034.7-19659.3] | 1.71 | < 0.0001 |
| let-7f | **0.51** [0.01-56.3] | **11.2** [2.4-33.2] | 22.09 | < 0.001 |
| miR-335 | **21.9** [0.02-456.6] | **318.0** [2.2-1335.4] | 14.54 | < 0.001 |
| miR-889 | **0.87** [0.01-17.2] | **10.5** [0.02-32.2] | 12.04 | < 0.001 |
| miR-362-3p | **1.6** [0.02-24.0] | **12.2** [1.1-22.0] | 7.68 | < 0.001 |
| miR-493 | **5.4** [0.04-35.9] | **38.8** [0.02-75.5] | 7.22 | < 0.001 |
| miR-376c | **95.4** [1.2-585.5] | **601.9** [9.6-1207.4] | 6.31 | < 0.001 |
| miR-501-5p | **7.0** [0.02-99.4] | **30.8** [5.8-68.8] | 4.40 | < 0.001 |
| miR-15b | **53.1** [0.04-639.2] | **213.9** [43.9-611.3] | 4.03 | < 0.001 |
| miR-99b | **39.8** [0.36-252.8] | **150.9** [8.2-688.5] | 3.80 | < 0.001 |
| miR-193a-3p | **6.0** [0.36-52.2] | **19.1** [3.4-98.5] | 3.22 | < 0.001 |
| miR-152 | **305.1** [0.09-1234.6] | **714.1** [186.2-2156.0] | 2.34 | < 0.001 |
| miR-345 | **219.9** [5.0-827.9] | **413.4** [134.4-919.4] | 1.88 | < 0.001 |
| miR-655 | 4.5 [0.02-65.8] | **47.4** [1.1-129.1] | 10.47 | < 0.001 |
| miR-495 | **11.9** [0.04-261.0] | **186.2** [2.9-436.1] | 15.67 | 0.001 |
| miR-487b | **9.4** [0.04-134.9] | **126.6** [0.49-294.0] | 13.48 | 0.001 |
| miR-138 | **36.8** [0.04-914.1] | **287.8** [20.5-1186.1] | 7.83 | 0.001 |
| miR-337-5p | **8.1** [0.02-89.9] | **56.4** [0.10-225.8] | 6.94 | 0.001 |
| miR-142-3p | **36.8** [0.02-1078.5] | **178.6** [42.6-1489.2] | 4.85 | 0.001 |
| miR-199a-5p | **5.7** [0.02-44.2] | **18.7** [0.48-164.5] | 3.30 | 0.001 |
| miR-379 | **20.3** [0.02-188.3] | **65.8** [0.70-177.7] | 3.24 | 0.001 |
| miR-130a | **68.1** [0.02-613.6] | **220.1** [13.4-487.9] | 3.23 | 0.001 |
| miR-103 | **54.4** [0.04-414.9] | **128.5** [48.2-282.9] | 2.36 | 0.001 |
| miR-218 | **380.2** [25.5-1247.1] | **661.7** [139.2-2216.0] | 1.74 | 0.001 |
| miR-382 | **1.5** [0.02-237.0] | **65.7** [0.02-243.9] | 42.90 | 0.002 |
| miR-323-3p | **13.3** [0.07-200.5] | **188.0** [1.7-375.7] | 14.18 | 0.002 |
| miR-485-3p | **3.8** [0.02-68.6] | **32.9** [0.39-127.0] | 8.68 | 0.002 |
| miR-660 | **199.0** [0.04-1287.4] | **503.8** [168.4-1032.7] | 2.53 | 0.002 |
| miR-125a-5p | **439.4** [22.1-1652.4] | **785.6** [148.1-2754.2] | 1.79 | 0.002 |
| miR-339-3p | **58.2** [1.5-188.0] | **81.7** [37.2-156.7] | 1.40 | 0.002 |
| miR-532-5p | **172.2** [0.36-2277.8] | **634.1** [113.7-1435.3] | 3.68 | 0.003 |
| miR-410 | **8.3** [0.04-83.5] | **88.0** [0.36-230.6] | 10.67 | 0.003 |
| miR-28-5p | **45.8** [0.04-244.0] | **107.0** [33.6-254.7] | 2.34 | 0.003 |
| miR-758 | **2.0** [0.02-29.3] | **13.9** [0.27-39.2] | 6.78 | 0.004 |
| miR-365 | **42.8** [0.41-349.7] | **89.9** [6.0-360.7] | 2.10 | 0.004 |
| miR-539 | **68.6** [0.04-385.1] | **456.8** [0.88-948.9] | 6.66 | 0.005 |
| miR-340 | **38.0** [0.13-266.5] | **101.0** [5.1-198.0] | 2.66 | 0.005 |
| miR-411 | **126.8** [3.3-590.5] | **317.1** [6.8-756.5] | 2.50 | 0.005 |
| miR-362-5p | **18.5** [0.03-312.0] | **86.0** [13.2-325.6] | 4.65 | 0.006 |
| miR-494 | **63.2** [1.5-567.0] | **213.5** [8.6-606.5] | 3.38 | 0.006 |
| miR-26b | **683.6** [0.02-1992.0] | **1180.5** [115.6-2788.1] | 1.73 | 0.007 |
| miR-92a | **87.9** [0.04-844.1] | **308.9** [27.0-698.1] | 3.51 | 0.008 |
| miR-25 | **33.2** [0.02-262.1] | **81.1** [24.6-212.1] | 2.44 | 0.008 |
| miR-433 | **7.5** [0.04-106.5] | **89.7** [0.02-290.2] | 11.95 | 0.009 |
| miR-370 | **28.7** [0.08-232.6] | **140.3** [0.02-302.8] | 4.88 | 0.009 |
| miR-149 | **130.6** [2.4-890.9] | **377.9** [15.9-3013.8] | 2.89 | 0.009 |
| miR-374a | **235.8** [0.07-732.8] | **338.4** [90.9-662.1] | 1.43 | 0.009 |
| miR-193a-5p | **16.9** [0.02-168.9] | **49.4** [1.1-219.7] | 2.92 | 0.010 |
| miR-148b | **6.8** [0.02-34.6] | **14.3** [0.6-29.5] | 2.12 | 0.010 |
| miR-127-3p | **119.7** [1.9-1532.9] | **846.6** [10.6-2671.2] | 7.07 | 0.011 |
| miR-455-5p | **49.7** [0.04-816.2] | **94.2** [22.0-596.8] | 1.90 | 0.015 |
| miR-222 | **4202.3** [2.4-14331.2] | **5637.3** [1592.5-44561.2] | 1.34 | 0.015 |
| miR-99a | **638.0** [0.02-6728.3] | **1164.7** [500.1-3923.4] | 1.83 | 0.016 |
| miR-100 | **887.6** [0.12-8235.6] | **1613.2** [704.5-5658.2] | 1.82 | 0.019 |
| miR-361-5p | **13.1** [0.02-73.4] | **27.7** [10.1-73.2] | 2.11 | 0.021 |
| miR-708 | **133.9** [0.02-717.5] | **325.8** [70.9-414.1] | 2.43 | 0.023 |
| miR-140-5p | **1432.1** [4.2-3696.0] | **2112.8** [601.3-30177.6] | 1.48 | 0.023 |
| miR-20b | **24.0** [0.04-232.8] | **43.6** [14.6-118.8] | 1.82 | 0.025 |

^a^Median expression in MPNSTs / Median expression in plexiform neurofibromas

^b^Mann and Whitney's U Test

^c^Median [range] of miRNAs levels

**Additional file 1: Table S4.** MRNA levels of four miRNAs processing machinery component genes (*DICER*, *DROSHA*, *DGCR8* and *AGO2*32) in nine dermal neurofibromas, 41 plexiform neurofibromas, and 15 MPNSTs.

| **mRNAs** | **Dermal neurofibromas (n=9)** | **Plexiform neurofibromas**  **(n=41)** | **Fold change**^a^ | ***P^b^*** | **MPNSTs**  **(n=15)** | **Fold change**^c^ | ***P^b^*** |
| --- | --- | --- | --- | --- | --- | --- | --- |
| *DICER* | 1.00 [0.22 – 7.54]^d^ | 1.31 [0.02 – 4.13] | 1.3 | NS | 1.09 [0.01 – 7.34] | 0.8 | NS |
| *DROSHA* | 1.00 [0.60 – 1.56] | 0.75 [0.39 – 1.66] | 0.8 | NS | 1.28 [0.41 – 2.87] | 1.7 | NS |
| *DGCR8* | 1.00 [0.62 – 1.26] | 1.00 [0.39 – 2.08] | 0.7 | NS | 0.45 [0.18 – 2.32] | 0.6 | NS |
| *AGO2*32 | 1.00 [0.93 – 1.45] | 0.74 [0.10 – 4.06] | 0.7 | NS | 1.02 [0.01 – 7.50] | 1.4 | NS |

^a^Median expression in plexiform neurofibromas / Median expression in dermal neurofibromas

^b^Mann-Whitney's U Test

^c^Median expression in MPNSTs / Median expression in plexiform neurofibromas

^d^Median [range] of miRNAs levels

NS, not significant. MRNAs were considered as significantly differentially expressed between plexiform and dermal neurofibromas, and between MPNSTs and plexiform neurofibromas when the *P*-value of the non-parametric comparison Mann-Whitney test was less than 0.01 (*P* < 0.01).
